# Surgical outcomes of unilateral recession and resection in intermittent exotropia according to forced duction test results

**DOI:** 10.1371/journal.pone.0200741

**Published:** 2018-07-26

**Authors:** Seonghwan Kim, Hee Kyung Yang, Jeong-Min Hwang

**Affiliations:** Department of Ophthalmology, Seoul National University College of Medicine, Seoul National University Bundang Hospital, Seongnam, Korea; Eye Research Center, Farabi Eye Hospital, ISLAMIC REPUBLIC OF IRAN

## Abstract

**Purpose:**

To compare the surgical outcomes of unilateral lateral rectus recession–medial rectus resection (RR) according to forced duction test (FDT) results with those of conventional RR in intermittent exotropia.

**Methods:**

A total of 129 patients aged 3 to 10 years with intermittent exotropia who underwent RR between 2006 and 2011 were included. The operator compared the tension of the lateral rectus (LR) between both eyes. When FDT results were asymmetric, RR was performed on the eye with more LR tension. RR was performed on the nondominant eye when FDT results were symmetric. Patients were divided into two groups; one group (n = 64) underwent RR without FDT (RR group) and the other group (n = 65) underwent RR considering FDT results (RR-FDT group). Success, recurrence, reoperation rates and cumulative probabilities of success were evaluated in both groups. Surgical outcome was considered satisfactory if the distance deviation in the primary position was between ≤ 10 PD of exophoria/tropia and ≤ 10 PD of esophoria/tropia. Recurrence was defined as an alignment of > 10 PD of exophoria/tropia, and overcorrection defined as > 10 PD of esophoria/tropia. Reoperation for recurrence was recommended for constant exotropia ≥ 14 PD at distance.

**Results:**

The total follow-up periods were 4.4±2.3 years in the RR group, and 3.9±2.0 years in the RR-FDT group (P = .310). In the RR group, 50 patients (78.1%) were successful, 13 patients (20.3%) had recurrence, and 1 patient (1.6%) had overcorrection at 2 years after surgery. In the RR-FDT group, 58 patients (89.2%) were successful, 5 patients (7.7%) had recurrence, and 2 patients (3.1%) were overcorrected. The recurrence rate at 2 years after operation was significantly lower in the RR-FDT group (P = .045). Recurrence rates during the follow-up period were 5.6% per person-year in the RR group and 2.7% per person-year in the RR-FDT group. Reoperation for recurrence was performed on 7 patients (10.8%) in the RR-FDT group and 16 patients (25.0%) in the RR group (P = .035). Postoperative sensory outcomes were similar between both groups.

**Conclusions:**

The forced duction test was useful in reducing the risk of recurrence at 2 years after surgery when RR was performed on the eye with more passive tension of the LR. Intraoperative FDT may be considered to choose which eye to operate on when planning RR in intermittent exotropia.

## Introduction

Intermittent exotropia (IXT) is the most common form of exotropia and is more prevalent among Asians [1–3]. Recurrence after exotropia surgery is frequently observed and the low success rate often frustrates the operator. Exotropic drift is common after surgical management of IXT and several studies have attempted to reduce the rate of recurrence and reoperation by revealing the largest angle of exodeviation preoperatively, or by augmenting the amount of surgery [4–10].

The pathophysiology of exotropia is poorly understood, however, innervational factors such as impaired ability to maintain fusion and alignment are considered to be one of the main reasons of frequent recurrence after surgery [11]. On the other hand, mechanical factors have also been suggested to affect abnormal positions of the eye, such as size and shape of the orbit and globes, volume and viscosity of retrobulbar tissue and functioning of the muscles as determined by their insertion, length and elasticity [12].

The forced duction test (FDT) is a simple and useful method for diagnosing the presence of mechanical force or restriction of the extraocular muscles (EOM) and can also be used to assess the tension or laxity of the lateral rectus muscle (LR) in IXT [13, 14]. To date, the surgical outcome of exotropia surgery considering intraoperative assessment of relative LR tension has not been previously investigated. Therefore, we investigated the surgical outcomes of unilateral lateral rectus recession–medial rectus resection (RR) performed in accordance with FDT results of the LR, and compared the results with those of conventional RR.

## Materials and methods

A retrospective review of medical records was performed on 129 consecutive patients of age 3 to 10 who underwent RR for IXT by one surgeon (J.-M.H.) between 2006 and 2011. Patients were divided into two groups; one group underwent RR without performing the FDT (RR group) and the other group underwent RR considering FDT results (RR-FDT group). Since 2009, we assessed the relative tension of both LR during surgery for IXT under general anesthesia, therefore, patients in the RR group were recruited before 2009 and those of the RR-FDT group were recruited since 2009. If intraoperative FDT results were symmetric, unilateral LR recession and medial rectus resection (RR) was performed on the nondominant eye. If FDT results were asymmetric, unilateral RR was performed on the eye with more passive tension of the LR. The eye selected for operation was randomly chosen if unilateral dominancy or asymmetric FDT were not obvious.

The minimum required follow-up period after surgery was 24 months, except for patients who required reoperation within 24 months after the first surgery. Patients with true divergence excess-type exotropia, congenital anomalies, neurologic disorders, paralytic strabismus, histories of strabismus surgery, moderate to severe amblyopia, coexisting ocular diseases other than strabismus, angle of exodeviation over 40 prism diopters (PD) were excluded from our study. Patients with dissociated vertical deviation, A or V patterns, or oblique muscle overactions requiring surgery were also excluded. Ethics approval was provided by the Institutional Review Board of Seoul National University Bundang Hospital. All aspects of the research protocol were in compliance with the Declarations of Helsinki. All data were fully anonymized before access and the IRB ethics committee waived the requirement for informed consent.

### Preoperative ophthalmologic examination

A prism and alternate cover test with accommodative targets for fixation at 0.3 m and 6 m was performed. An additional near measurement was obtained after 1 hour of monocular occlusion of the habitually deviating eye, and another postocclusion near measurement was obtained with an additional +3.00 diopters (D) sphere over each eye prior to allowing the patient to regain binocular fusion. The presence of fixation dominance was determined with repeated examinations of the cover-uncover test. Refractive errors were determined by cycloplegic refraction with cyclopentolate hydrochloride 1% and analyzed as spherical equivalent values. For patients with myopia of -1.00 D or more, spectacles of full cycloplegic refraction were prescribed. In patients with hyperopia > +3.00 D, spectacles of approximately +1.00 to +1.50 D less than the full cycloplegic refraction were given. Also, spectacles were prescribed for patients with anisometropia, defined as a spherical or cylindrical difference of > 1.50 D between both eyes. Amblyopia was defined as a difference of 2 lines or more between monocular visual acuities, and only mild amblyopia with the best corrected visual acuity of the worse eye > 20/40 were included. Lateral incomitance was defined as ≥ 5 PD change in the lateral gaze from the primary position. An A pattern was defined as an increase of 10 PD or more of exodeviation at downgaze compared with upgaze, and V pattern was defined as an increase of 15 PD or more of exodeviation at upgaze compared with downgaze. Sensory status was evaluated with the Randot stereoacuity test at distance and near. Stereoacuity of ≤ 100 seconds or arc (arcsec) was defined as good.

### Intraoperative procedures

All surgeries were performed under general anesthesia by one surgeon (J-MH) according to the same surgical table (Table 1). From 2006 to 2008, RR was done conventionally without FDT on the nondominant eye for the patients with a dominant fixating eye. Since 2009, FDT was performed on both eyes to measure the degree of relative LR tension under general anesthesia before starting surgery. By grasping the conjunctiva with toothed forceps and moving the eyeball toward adduction, the operator compared the tension of LR between the two eyes. When FDT results were asymmetric in both eyes, RR was performed on the eye with more LR tension. RR was performed on the nondominant eye when FDT results were symmetric. RR was performed according to the Wright formula [15] based on the largest angle of preoperative deviation measured during distance or near fixation; LR recession was done based on the deviation measured at distance, and MR resection based on the deviation at near.

**Table 1:** Surgical table.

	Deviation at distance, PD	LR recession, mm	Deviation at near, PD	MR resection, mm
RR	15	4	15	3
	20	5	20	4
	25	6	25	5
	30	7	30	5.5
	35	7.5	35	6
	40	8	40	6.5

PD = prism diopters, LR = lateral rectus muscle, MR = medial rectus muscle, RR = lateral rectus muscle recession and medial rectus resection

### Postoperative measurements

Postoperative alignment at distance in the primary position was measured at 1, 6, 12 and 24 months postoperatively and afterwards. Patients with diplopia associated with postoperative esotropia were managed with alternating full-time patching for 1–4 weeks until diplopia resolved. If esotropia still existed with alternate patching for 4 weeks, cycloplegic refraction was performed, and hyperopia > +1.00 D was corrected. Patients without hyperopia of +1.00 D were prescribed base-out Fresnel press-on prisms (3M Health Care, St Paul, Minnesota, USA) to facilitate constant fusion. When it became evident that prisms would have to be worn for several months, prisms incorporated into regular spectacles were prescribed. Surgical outcome was considered satisfactory if the distance deviation in the primary position was between ≤ 10 PD of exophoria/tropia and ≤ 10 PD of esophoria/tropia. Recurrence was defined as an alignment of > 10 PD of exophoria/tropia, and overcorrection defined as > 10 PD of esophoria/tropia. Reoperation for overcorrected patients was performed if esotropia of > 20 PD persisted or increased for 6 months after surgery. Reoperation for recurrent or residual exotropia was recommended for constant exotropia ≥ 14 PD at distance, despite treatment by nonsurgical means, such as part-time occlusion or minus-lens therapy in most patients. Improved stereopsis was defined as a decrease of 2 octaves or more seconds of arc, and decreased stereopsis was defined as an increase of more than 2 octaves or more seconds of arc [16].

### Main outcome measures

The outcome measures were long-term recurrence rates and overcorrection based on postoperative alignment at distance and improvement in stereopsis.

### Statistical analysis

Statistical analyses were performed using SPSS software for Windows v 22.0 (SPSS, Chicago, Illinois, USA). The independent t test, χ^2^ test, and Fisher’s exact test were used to compare the patient’s characteristics and the surgical outcomes. Kaplan-Meier life-table analysis was used to compare the long-term cumulative probability of recurrence between the RR group and RR-FDT group. P < .05 was considered statistically significant.

## Results

### Patient demographics

Among the 129 patients with IXT who underwent RR, 64 patients received conventional RR (RR group) and 65 patients underwent RR considering FDT results (RR-FDT group). The preoperative patient characteristics were not significantly different between the two groups (Table 2) except for lateral incomitance, which was more common in the RR-FDT group (23.1% vs 9.4%) compared to the RR group. FDT results were symmetric in 28 patients (43.1%) and asymmetric in 37 patients (56.9%). In the RR-FDT group, lateral incomitance was not significantly different according to the symmetry of FDT results (25.0% vs 21.6%, P = .749).

**Table 2:** Patient characteristics in children with intermittent exotropia who received unilateral lateral rectus recession and medial rectus resection without performing the forced duction test (RR Group) and those who received unilateral lateral rectus recession and medial rectus resection considering forced duction test results (RR-FDT Group).

Variable	RR group (n = 64)	RR-FDT group (n = 65)	P value
Sex (M:F)	31:33	31:34	.947^†^
Age of onset (y)	3.3 ± 2.5 (0.3–8.0)	3.7 ± 2.4 (0.7–8.0)	.278^‡^
Age at surgery (y)	6.0 ± 2.3 (3–10)	6.7 ± 2.4 (3–10)	.291^‡^
Duration of follow-up (y)	4.4 ± 2.3 (0.5–9.0)	3.9 ± 2.0 (0.5–8.0)	.310^‡^
Type of exodeviation			
Basic	54 (84.4%)	51 (78.5%)	.745^†^
Simulated divergence excess	5 (7.8%)	6 (9.2%)	.215^†^
Convergence insufficiency	5 (7.8%)	8 (12.3%)	.109^†^
Unilateral dominancy	26 (40.6%)	36 (55.4%)	.093^†^
Lateral incomitance	6 (9.4%)	15 (23.1%)	.035^†^
Maximum preoperative deviation			
Distance (PD)	28.9 ± 6.3 (15–40)	26.0 ± 7.3 (15–40)	.321^‡^
Near (PD)	29.8 ± 6.8 (15–40)	28.2 ± 8.2 (15–40)	.447^‡^
Amount of surgery			
Lateral rectus recession (mm)	6.58 ± 0.91 (4.0–8.0)	6.11 ± 1.25 (4.0–8.0)	.312^‡^
Medial rectus resection (mm)	5.52 ± 0.81 (3.0–6.5)	5.10 ± 1.12 (3.0–6.5)	.384^‡^

Abbreviation: RR, unilateral lateral rectus recession-medial rectus resection; FDT, forced duction test; PD, prism diopters

Parameters are given as mean with standard deviations (range).

^†^ Chi square test

^‡^ Independent t test

### Surgical outcome

**Fig 1:**
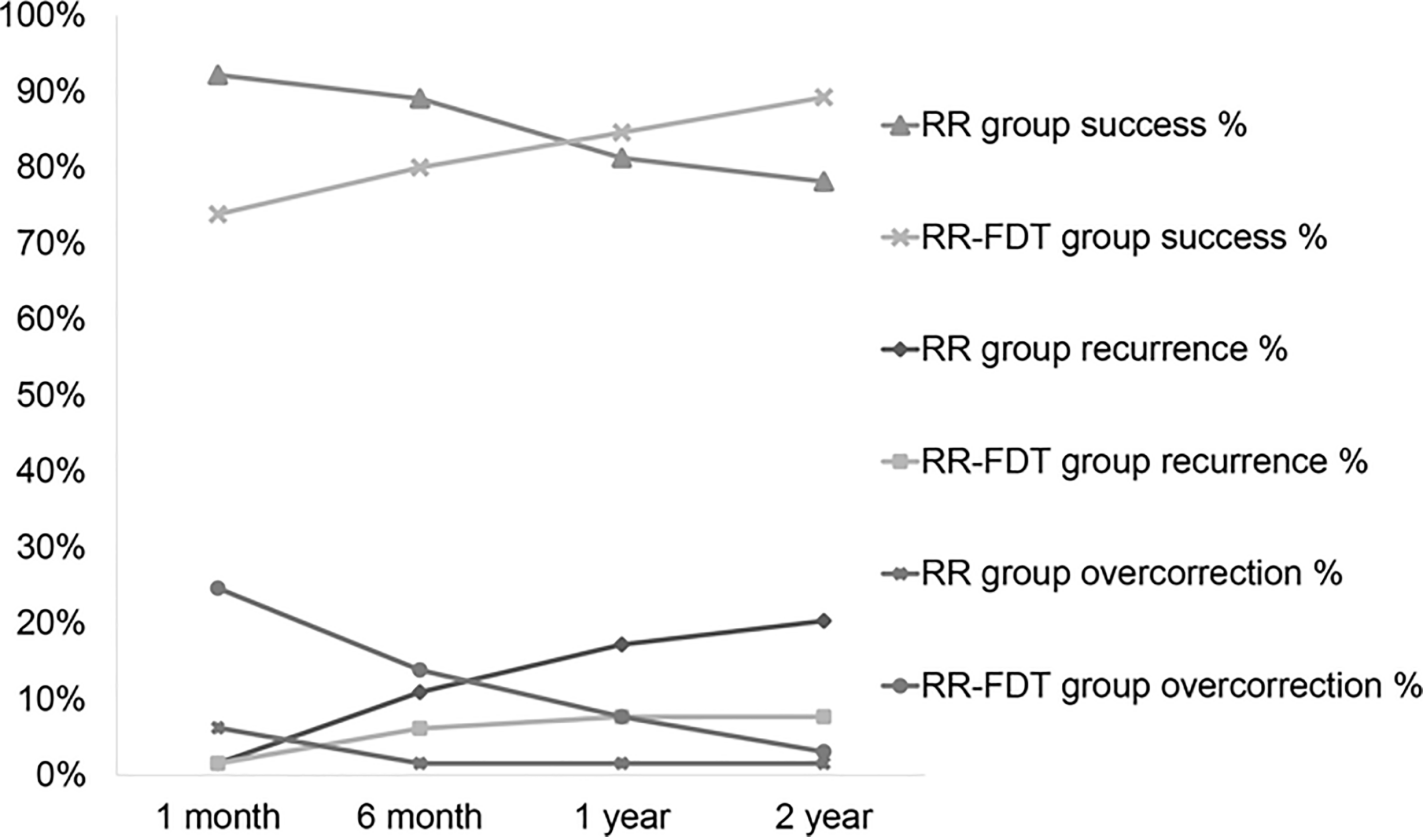
Surgical outcomes at 1 month, 6 months, 1 year, and 2 years in patients who received unilateral lateral rectus recession and medial rectus resection without performing the forced duction test (RR group) and those who received unilateral lateral rectus recession and medial rectus resection considering forced duction test results (RR-FDT group).

In the RR group, 50 patients (78.1%) were successful, 13 patients (20.3%) had recurrence, and 1 patient (1.6%) had overcorrection at 2 years after surgery (Table 3). In the RR-FDT group, 58 patients (89.2%) were successful, 5 patients (7.7%) had recurrence, and 2 patients (3.1%) were overcorrected at 2 years postoperatively (Table 3, Fig 1). The recurrence rate at 2 years after operation was significantly lower in the RR-FDT group (20.3% vs 7.7%) (P = .045).

**Table 3:** Recurrence and overcorrection rates up to 2 years in patients who received unilateral lateral rectus recession and medial rectus resection without performing the forced duction test (RR group) and those who received unilateral lateral rectus recession and medial rectus resection considering forced duction test results (RR-FDT group).

Group		Recurrence				Overcorrection			
		1 month	6 months	1 year	2 years	1 month	6 months	1 year	2 years
RR group (n = 64)	No.	1	7	11	13	4	1	1	1
	%	1.6	10.9	17.2	20.3	6.3	1.6	1.6	1.6
RR-FDT group (n = 65)	No.	1	4	5	5	16	9	5	2
	%	1.5	6.2	7.7	7.7	24.6	13.8	7.7	3.1
P-value		1.000^†^	.364^†^	.117^‡^	.045^‡^	.004^†^	.009^†^	.107^†^	.506^†^

Abbreviation: RR, unilateral lateral rectus recession-medial rectus resection; FDT, forced duction test

^†^ Fisher’s exact test

^‡^ Chi square test

Twenty-three patients eventually required reoperations for recurrence during the follow-up period; 16 patients (25.0%) in the RR group, 7 patients (10.8%) in the RR-FDT group. The reoperation rate was significantly lower in the RR-FDT group (P = .035). The total follow-up periods were 4.4 ± 2.3 years in the RR group, and 3.9 ± 2.0 years in the RR-FDT group, which were not significantly different (P = .310). Recurrence rates during the total follow-up period were 5.6% per person-year in the RR group and 2.7% per person-year in RR-FDT group.

Until 6 months after surgery, the overcorrection rate was significantly higher in the RR-FDT group (P = .009). However, 14 of 16 initially overcorrected patients (87.5%) in the RR-FDT group had less than 10 PD of esotropia at the last follow-up examination. No overcorrected patients from both groups required reoperation.

In the RR-FDT group, eight patients (12.3%) received RR on their dominant eye after FDT, which was the major difference from the conventional method. Of the 8 patients, 7 patients (87.5%) were successful, no patient had overcorrection and 1 patient (12.5%) showed recurrence at 2 years after the operation, who eventually received reoperation (Table 4). In the RR-FDT group, the overall success rates at 2 years were not significantly different in those who received surgery on the dominant eye (87.5%) compared to those who received surgery on the nondominant eye (78.9%) (P = .491).

**Table 4:** Recurrence and overcorrection rates at postoperative follow-up examinations up to 2 years in 8 patients who underwent unilateral lateral rectus recession and medial rectus resection on the dominant eye based on forced duction test results.

		1 month	6 months	1 year	2 years
Recurrence	No.	0	1	1	1
	%	0	12.9	12.9	12.9
Overcorrection	No.	4	3	2	0
	%	50	37.5	25	0

Abbreviation: RR, unilateral lateral rectus recession-medial rectus resection; FDT, forced duction test

### Binocular function

Among all patients, 45 of 64 patients in the RR group and 48 of 65 patients in the RR-FDT group were capable to test stereoacuity. Good stereoacuity was present in 75.6% (34/45) in the RR group and 68.8% (33/48) in the RR-FDT group (P = .465, χ^2^ test) at the last follow-up examination. Improvement in stereopsis was found in 28.9% (13/45) in the RR group and 25.0% (12/48) in the RR-FDT group, which was not significantly different (P = .672). Stationary stereopsis was found in 60.0% (27/45) in the RR group and 62.5% (30/48) in the RR-FDT group (P = .805). Decreased stereopsis was found in 11.1% (5/45) in the RR group and 12.5% (6/48) in the RR-FDT group (P = .836). Consequently, postoperative sensory outcomes were similar between both groups.

## Discussion

Our study evaluated the surgical outcomes of RR considering LR tension as the major factor in deciding which eye to operate on, and compared the results with conventional RR performed on the nondominant eye. The RR-FDT group showed less recurrence and reoperation rates compared to conventional RR without increasing the rate of long-term overcorrection.

In previous studies, the strategy of planning the surgical dose based on the largest angle ever measured [17] and setting the target as a small to moderate angle of initial overcorrection [5, 18] has been tried to overcome postsurgical exodrift of IXT. Recently, Kim et al. [8] reported that augmented bilateral LR recession resulted in more successful surgical outcomes and lower recurrence rates. However, to the best of our knowledge, there has been no study investigating the surgical outcomes of RR considering the tension of EOM in IXT. Tension of the EOM may cause resistance of the eyeball in moving to the opposite direction, and in such cases, decreasing the tension by recessing the muscle might be beneficial in reducing recurrence and improving success rates. Conversely, surgery without considering the tension of the EOM may be responsible for the high rate or failure in exotropia surgery. Thus, we planned our surgery to consider the tension of EOM and to compare the results with conventional methods based solely on the dominancy of exotropia.

There may be several explanations for the reason of tension found on FDT during general anesthesia. Longstanding strabismus may result in fibrosis of the LR tendon and cause a certain amount of restriction. The tension of EOM during general anesthesia may also vary depending on the depth of anesthesia and use of muscle relaxants. However, future experimental studies should be conducted to prove our assumptions and clarify the pathophysiological changes related to different FDT results.

Jeoung et al. [19] reported that RR resulted in a better outcome than bilateral lateral rectus recession in exotropia with a dominant eye and RR was performed on the nondominant eye conventionally. However, our study revealed that when FDT results are asymmetric, RR performed on the eye with more LR tension may be beneficial even if it was the dominant eye. Among patients in the RR-FDT group, only 8 patients (12.3%) received RR on their dominant eye after FDT, and the overall success rates at 2 years were comparable to those who received surgery on the nondominant eye. This implies that it is safe to perform surgery on the dominant eye if there is more LR tension. However, due to the small number of patients with more LR tension on the dominant eye, a larger study population is necessary to clarify the efficacy or superiority of exotropia surgery based on FDT results.

Regarding overcorrection, the high rate of initial overcorrection in the RR-FDT group slowly decreased after 2 years. The overcorrection rate was higher in the RR-FDT group up to 1 year, but no significant difference was found after 2 years in both groups. Lateral incomitance was more common in the RR-FDT group. As surgery was done based on the deviation measured in the primary position, this might overestimate the actual deviation, and overcorrection of exotropia might occur after recession of the tense LR. This could be one of the explanations of the high rate of immediate postoperative overcorrection in the RR-FDT group. However, lateral incomitance was not more frequent in patients with asymmetric EOM tension between both eyes.

This study has a few limitations. First, only children with exodeviations of 40 PD or less were included in the study. Children with more than 40 PD of exodeviation were mostly related to systemic conditions (e.g. severe general illness, facial malformation, genetic disease, or trauma) and were excluded. Second, FDT is a subjective examination of which the interpretation of results highly depends on the examiner. Several previous investigators have developed methods to quantify the passive forced duction test for extraocular rectus muscles [20–22]. Stephens and Reinecke [22] developed a perilimbal suction cup placed on the globe and attached to a force displacement transducer. Metz [21] also developed a strain gauge force transducer, and Rosenbaum and Myer [20] used a spring dynamometer which measured the grams of force required to displace the eye from its rest position into the various fields of rotation of the globe. However, none of those methods are clinically available. As the absolute tension of EOM is subject to the depth of anesthesia and may be highly variable between examiners, we compared the relative tension of both LR to decide which eye to operate on [23]. Further prospective studies are required to confirm the results from our study. Lastly, the two groups were not recruited from the same period since FDT was performed only after 2009. Nevertheless, the same surgeon operated on both groups and there was no change in the surgical technique, suture materials and postoperative management that may affect surgical outcomes.

In conclusion, RR performed on the eye with more passive tension of the LR resulted in successful alignment and lower recurrence at 2 years after surgery. Intraoperative FDT might be considered when planning RR in intermittent exotropia.
